# Optimizing pediatric surgical analgesia: recent trends in regional anesthesia

**DOI:** 10.1016/j.bjane.2025.844695

**Published:** 2025-10-25

**Authors:** Mariana Fontes Lima Neville, Vinícius Caldeira Quintão, John Hagen, Liana Maria Torres de Araújo Azi

**Affiliations:** a Universidade Federal de São Paulo, Escola Paulista de Medicina, Hospital São Paulo, São Paulo, SP, Brazil; b Universidade de São Paulo, Faculdade de Medicina, Departamento de Pediatria, São Paulo, SP, Brazil; c Universidade de São Paulo, Faculdade de Medicina, Disciplina de Anestesiologia, São Paulo, SP, Brazil; d Memorial Sloan Kettering Cancer Center, Anesthesiology & Critical Care Medicine, New York, USA; e Universidade Federal da Bahia, Salvador, BA, Brazil

Regional anesthesia increasingly occupies a central role in pediatric anesthesia. Most children undergoing surgical procedures can benefit from a regional technique.^1^^,^^2^ Assessing and managing pain in childhood remains a constant challenge, particularly in preverbal or nonverbal children. An agitated emergence may reflect pain, emergence delirium, anxiety, hunger, or discomfort. Therefore, implementing effective, long-lasting analgesic strategies with minimal side effects is essential for safe and smooth postoperative recovery.

Clinical experience and scientific evidence consistently highlight the clear advantages of regional anesthesia in pediatric patients: reduced intraoperative opioid use, smoother emergence, shorter recovery room stays, prolonged analgesia, and a lower incidence of postoperative complications such as paralytic ileus and atelectasis. Beyond these direct clinical benefits, greater satisfaction is also observed among the child, family members, and the multidisciplinary perioperative care team.^3^, ^4^, ^5^

Over the past decades, one of the most remarkable advances has undoubtedly been the incorporation of ultrasonography into regional anesthesia practice. Ultrasound has transformed the landscape of pediatric anesthesia by enabling real-time visualization of anatomical structures — such as nerves, vessels, and fascial planes — and monitoring of local anesthetic spread. This approach has enhanced safety, reduced the risk of inadvertent punctures, minimized the need for large volumes, and expanded the repertoire of available blocks, particularly fascial plane blocks. As a result, technical success rates have significantly increased, with fewer needle passes, faster onset, and longer-lasting analgesia.^6^^,^^7^

This technological progress has been accompanied by robust evidence demonstrating the safety of regional anesthesia in children. For many years, it was believed that performing blocks under general anesthesia might mask early signs of neural injury. However, prospective multicenter studies — such as the Pediatric Regional Anesthesia Network (PRAN) and the Association des Anesthésistes Réanimateurs Pédiatriques d'Expression Française (ADARPEF) — have consistently documented the safety of regional anesthesia in children, including almost 160,000 blocks without evidence of permanent neurologic sequelae.^4^^,^^5^^,^^8^ In addition, a recent study using magnetic resonance imaging to measure the distances between neural structures and the epidural canal demonstrated substantial safety margins for thoracic and lumbar punctures in pediatric patients, strengthening the anatomic evidence for the safety of these techniques.^9^

Expanding from safety to efficacy, abdominal wall blocks have become integral to modern pediatric anesthesia. The most commonly used include the transversus abdominis plane (TAP), quadratus lumborum (QL), and rectus sheath blocks, with several technical variations. These techniques have become routine in surgeries such as herniorrhaphies, appendectomies, and urological procedures.

Recent studies comparing the analgesic efficacy of TAP and QL blocks suggest that QL may be superior in reducing intraoperative opioid consumption and postoperative pain scores.^10^^,^^11^ In a double-blind clinical trial, Mutlu and colleagues observed that children receiving QL blocks had lower pain scores and reduced remifentanil requirements compared with the TAP group.^12^

The QL block can be performed using different approaches — lateral (QL1), posterior (QL2), and anterior or transmuscular (QL3) — which vary in complexity and patterns of anesthetic spread. In a randomized study of 120 children, Arun et al. compared the three approaches and demonstrated that the anterior approach resulted in lower fentanyl consumption, longer analgesic duration, and greater parental satisfaction.^13^ These findings emphasize that approach selection should be individualized, taking into account the surgical procedure, operator experience, and patient profile.

Beyond the abdominal wall, thoracic approaches such as the erector spinae plane (ESP) block has emerged as a versatile and safe alternative applicable to abdominal, thoracic, and cardiac procedures. When performed under ultrasound guidance, ESP is relatively straightforward, and its anatomical target lies distant from critical structures such as the pleura and spinal cord.

A recent meta-analysis including nine clinical trials and 507 patients showed that the ESP block provides analgesia comparable to caudal block, with a lower incidence of urinary retention.^14^ This finding is particularly relevant in short procedures and ambulatory patients, in whom early mobilization and discharge are desirable.

In a broader context, a network meta-analysis by Wegner et al. on pediatric cardiac surgery demonstrated that the transversus thoracic muscle plane block (TTPB) and thoracic paravertebral block are among the most effective techniques for post-sternotomy analgesia, significantly reducing opioid consumption and extubation time.^15^ These results expand the concept of regional anesthesia beyond the abdominal wall, integrating thoracic and paravertebral blocks as key components of pediatric enhanced recovery after surgery (ERAS) protocols.

For upper-limb procedures, the infraclavicular block has become the technique of choice for forearm, wrist, and hand surgeries. Recently, Yayik et al. compared the lateral sagittal and costoclavicular approaches, finding significantly shorter procedure times with the latter, without differences in analgesic efficacy or safety.^16^ This is particularly relevant in pediatric practice, where procedural agility and predictability directly influence anesthetic workflow efficiency and patient stability.

In addition to perineural and fascial plane blocks, other analgesic strategies are gaining ground in modern pediatric anesthesia. Intraperitoneal instillation of local anesthetic is a simple, quick, and low-risk technique. Moen and colleagues compared bupivacaine combined with dexmedetomidine or magnesium sulfate in pediatric laparoscopic surgeries, and found lower pain scores and fewer rescue analgesic requirements with adjuvant use, without an increase in adverse effects.^17^ These findings reinforce the role of multimodal strategies and rational adjuvant use to optimize analgesia and accelerate postoperative recovery.

The advances described in these studies — from MRI-validated safety margins to novel block comparisons — represent significant progress in pediatric regional anesthesia. However, translating this evidence into practice requires thoughtful educational frameworks.

Recent international consensus work by Hagen et al. identified core pediatric regional anesthesia techniques that balance clinical effectiveness with accessibility, providing a structured, consensus-driven model for training programs.^18^ This framework builds on earlier conceptual work advocating for simplified, high-value blocks to improve adoption rates. Importantly, these guidelines should be viewed as structured starting points rather than rigid doctrine — encouraging practitioners to progress beyond foundational techniques as their skills and institutional capabilities evolve.

While the quadratus lumborum and erector spinae plane blocks featured prominently in these studies were not selected as core techniques — reflecting ongoing debates about reliability, complexity and standardization — their growing evidence base suggests they may represent a natural progression for practitioners who have mastered foundational blocks.

Facilitating this journey from foundational to advanced techniques, educational platforms like Baby Blocks (www.baby-blocks.com) exemplify how modern resources can bridge the gap between research and practice, offering structured learning pathways from basic to advanced techniques.^19^ These initiatives, combined with the growing evidence base summarized here, support the editorial's central message: optimizing pediatric surgical analgesia requires not only advancing techniques but also ensuring their thoughtful implementation through structured education that adapts to local contexts and evolves with emerging evidence.

In summary, pediatric regional anesthesia has evolved rapidly over the past two decades, driven by ultrasound incorporation, standardized multicenter registries, and a growing body of safety and efficacy evidence. The field is now entering a new era in which block selection is guided not only by anatomy but also by integration within multimodal enhanced recovery protocols.

Training in pediatric anesthesiology should therefore include mastery of these techniques, safe ultrasound use, and understanding of emerging evidence — such as that from recent studies on QL, ESP, and thoracic blocks. Optimizing surgical analgesia in children goes beyond pain reduction: it means improving outcomes, reducing opioid use, and humanizing anesthetic care.

## Conflicts of interest

JH serves as Editor-in-Chief of Baby Blocks. VCQ serves as contributor to Baby Blocks. No financial compensation interest is involved. All other authors declare no conflicts of interest.
